# Promotion of quality standard of herbal medicine by constituent removing and adding

**DOI:** 10.1038/srep03668

**Published:** 2014-01-13

**Authors:** Dan Yan, Junxian Li, Yin Xiong, Congen Zhang, Jiaoyang Luo, Yumei Han, Ruiling Wang, Cheng Jin, Hong Qian, Jiangyu Li, Lingling Qiu, Cheng Peng, Yuling Lin, Xueai Song, Xiaohe Xiao

**Affiliations:** 1 China Military Institute of Chinese Materia Medica, Military 302 Hospital, Beijing 100039, China; 2 National Center of Biomedical Analysis, Beijing 100850, China; 3 College of Traditional Chinese Pharmacy, Beijing University of Chinese Medicine, Beijing 100102, China; 4 Institute of Medicinal Plant Development, Chinese Academy of Medical Sciences, Beijing 100094, China; 5 Beijing Physical Examination Center, Beijing 100077, China; 6 Chengdu University of Traditional Chinese Medicine, Chengdu 610075, China; 7 These authors contributed equally to this work.

## Abstract

To identify major active constituents and measure their levels in a typical medicinal herb–*Rhizoma coptidis*, we applied the concept of removing and adding, taking inspiration from functional genetic methods. As this herb has bacteriostatic properties and is used to treat bacterial diarrhea, we examined the effects of individual constituents (berberine, palmatine, coptisine, epiberberine, jateorrhizine and columbamine) on the growth of *Shigella dysenteriae* with microcalorimetry. The removing and adding procedures revealed that berberine and coptisine were the main antibacterial constituents of *R. coptidis*, with bacteriostatic activities of 54.10% and 39.75%, respectively. The relative levels of berberine and coptisine in *R. coptidis* were 8.08%–31.92% and 4.05%–14.45%, respectively. On the basis of whole effect, the method of constituents removing and adding, coupled with a bioassay, is a useful strategy to identify the active constituents and measure their levels in herbal medicines, which may provide reference to other natural products.

The quality of a medicine provides an important foundation for its clinical efficacy. Unlike pharmaceutical drugs that contain specific active constituents and exhibit clear dose-dependent effects, herbal medicines are often derived from complex systems containing multiple components^1^,^2^. For a long time, the content detection of constituents is the main quality control (QC) method for herbal medicines. While as we know^3^,^4^,^5^,^6^,^7^,^8^, herbal medicines are prepared from complex organisms that contain multiple active constituents that act additively to elicit effects that greater than those of the individual components^9^,^10^,^11^. And the determination of indicators and their content level of the existing quality standard (QS) are often subjective without powerful evidences, which lead to the uncorrelation between the QS and clinical efficacy^12^,^13^,^14^. Consequently, herbal medicines are poorly accepted by the mainstream of medical community, including medical experts, clinical pharmacists, economists, sociologists, and patients^1^,^15^,^16^,^17^,^18^.

There are both genetic information (gene) and chemical information (constituent) existing in organisms. In the identification of functional genes, the discovery and validation of each gene is closely related to the strategy of gene targeting and related techniques, including knockout and knock-in methods^19^,^20^,^21^,^22^,^23^. This strategy has been successfully used to determine the contributions of functional genes within organisms, providing the basis for genetic screening and gene therapy (Supplementary Fig. S1a). By this inspiration (Supplementary Fig. S1b), methods to identify the active constituents and measure their levels in herbs, as complex organisms, can involve constituent removing (similar to knockout) and adding (similar to knock-in), which makes the setting of QS more well-founded related to the whole effect.

As the discovery of functional genes is closely related to the phenotypic analysis, the validation of active constituents in herbal medicines cannot be separated from the bioactivity detection. Microcalorimetry, an automatic and quantifiable method that measures changes in energy levels at the cellular, tissue, organ, or whole organism levels etc., has been used in physical, chemical, and biochemical fields^18^,^24^,^25^,^26^,^27^,^28^. Compared with conventional methods (e.g., microplate and turbidimetry assays), microcalorimetry can provide detailed information on the bacteriostatic effects of drugs in real-time, online, and high-throughput screening assays. Therefore, microcalorimetry is widely used to assess the bacteriostatic activity of drugs^29^,^30^,^31^,^32^,^33^.

*Rhizoma coptidis* (*R. coptidis*), an important herbal medicine to treat bacterial diarrhea, constipation, diabetes, and other major diseases in Asia, Africa, and Latin America for a long history^32^,^34^ (Supplementary Fig. S2), was studied in this research. There are a lot of protoberberine alkaloids in *R. coptidis*, of which the structure belongs to isoquinoline alkaloids. The structural differences of those alkaloids are mainly caused by different groups bound to C2, C3, C9 and C10 (Supplementary Fig. S3). But their contributions to the bacteriostatic activity of *R. coptidis* and the content levels have not been clearly specified. Thus, we put forward the strategy of constituent removing and adding, hoping to discover active constituents of *R. coptidis* inhibiting *Shigella dysenteriae* (*S. dysenteriae*) and their content limits. Specifically, we first removed the targeted constituents from *R. coptidis* to evaluate the inhibitory effects of the targeted constituent and its negative sample (samples lacking the targeted constituent) on the activity of *S. dysenteriae*, with unmodified *R. coptidis* extract as a reference sample. After confirming the identities of the active constituents, the targeted constituents were added into the negative samples individually to measure their bacteriostatic effects. We believe that the difficulty of characterizing the active constituents and measuring their levels in herbs can be overcome by applying the removing and adding strategy together with a bioassay. This method can provide an important reference for the establishment of QS related to the clinical efficacy. The experimental strategy used in this study is shown in Fig. 1.

## Results

### Constituent removing and chemical identification

A preparative thin layer chromatography plate (PTLCP) was used to achieve constituent removal. Each band on the plate corresponded to one constituent and was regarded as a positive sample (namely targeted constituent marked by letter M^+^). The rest of bands was defined as the corresponding negative sample (marked by letter M^−^).

Next, we used Ultra-performance liquid chromatography (UPLC) and quadrupole time-of-flight mass spectrometry (Q-TOF MS) to identify the constituent of each band, and to determine whether there were residual compounds in the corresponding negative sample. Using this method, the constituents in the positive samples were preliminarily identified (Supplementary Fig. S4) as berberine (BER), palmatine (PAL), coptisine (COP), epiberberine (EPI), jateorrhizine (JAT), and columbamine (COL), respectively. The levels of the removed constituents in the negative samples were nearly undetectable (Supplementary Fig. S4). Furthermore, the molecular structures of the removed constituents were not damaged by the removal procedure (Supplementary Fig. S5).

### Identification of the active constituents

The growth and metabolism of living organisms are accompanied by heat/energy production, which can be affected by pathological changes or the action of drugs. Therefore, it is possible to evaluate changes in microbial heat production in the presence or absence of different drugs using microcalorimetry. Accordingly, we determined the bioactivity of *R. coptidis* extract, removed samples, and added samples in terms of bacteriostasis.

**Figure 2:**
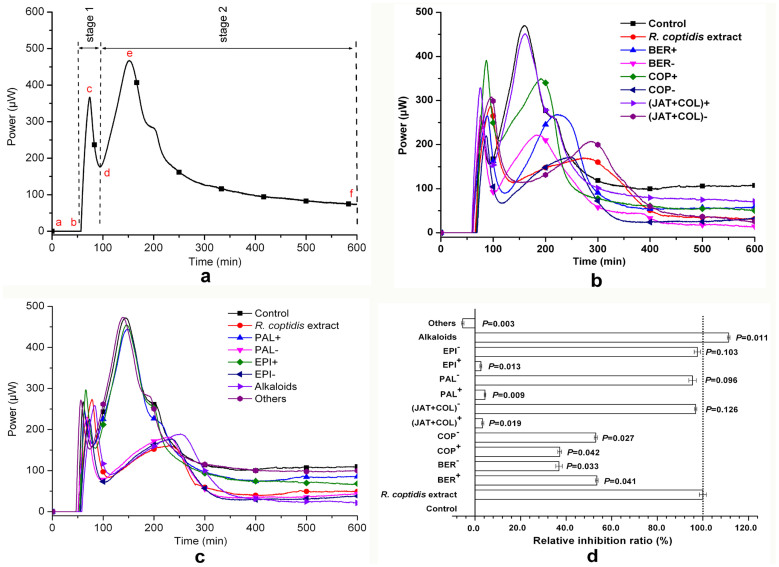
Results of identification of the active constituents. (a) Heat flow power-time (HFP-*t*) curve for control *S*. *dysenteriae* cultured in L.B. culture medium alone. (b, c) Effects of the *R*. *coptidis* extract, removed constituents, and negative samples on HFP-*t* curves of *S*. *dysenteriae* growth. (d) Contributions of the removed constituents and their corresponding negative samples to the bacteriostatic activity of *R*. *coptidis*. (b–d) Control: *S*. *dysenteriae* alone; reference: *R*. *coptidis* extract (0.8 mg/mL); removed samples: BER^+^, COP^+^, EPI^+^, PAL^+^, and (JAT + COL)^+^; negative samples: *R*. *coptidis* extract lacking COP (COP^−^), EPI (EPI^−^), PAL (PAL^−^) and JAT + COL combined (JAT + COL)^−^. The measurements of relative inhibition ratio were performed in triplicate and error bars represent standard error of the mean. *P* value compared to *R*. *coptidis* extract determined by two-way ANOVA.

The normal growth thermogenic curve for *S. dysenteria*e at 37°C is shown in Fig. 2a. The heat flow power-time (HFP-*t*) curve showed that the *S. dysenteriae* metabolic profile included two main stages (stages 1 and 2) and five phases, (lag phase [a–b], the first exponential growth phase [b–c], transition phase [c–d], the second exponential growth phase [d–e], and the decline phase [e–f]). The quantitative thermokinetic parameters of the HFP-*t* curve for *S. dysenteriae* growth could be delineated using the equation (1): 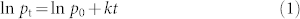
where *P*_0_ and *P*_t_ represent the heat flow power at time 0 or time (min), respectively. To test the reliability of the microcalorimetry, we repeated the experiment on eight occasions in untreated bacteria and obtained good reproducibility. We then quantified the following thermokinetic parameters from the HFP-*t* curves in the presence of difference concentrations of the samples: *p*_1_, *p*_2_, *t*_1_ and *t*_2_ (Table 1). PCA revealed that *k*_2_ and *t*_2_ explained 87% of the variation of samples, including the *R. coptidis* extract, removed samples, and added samples. Therefore, we focused on parameters *k*_2_ and *t*_2_ in this study.

Figures 2b, c illustrate the effects of *R. coptidis* (0.8 mg/mL) extract, removed constituents, and corresponding negative samples on the HFP-*t* curves of *S. dysenteriae*. We found that, compared with the control and the *R. coptidis* extract, the kinetics of the removed and negative samples showed marked variation (Supplementary Table S1).

Next, we calculated the inhibition ratio *I* as equation (2) and the relative inhibition ratio *RI* as equation (3) to quantify the contributions of the individual constituent to the bacteriostatic activity of *R. coptidis* extract. As shown in Fig. 2d, BER, COP, EPI, PAL, and JAT + COL removed inhibited the growth and metabolism of *S. dysenteriae*; of which BER and COP had the greatest effects. The bacteriostatic activities of BER^−^ and COP^−^ were significantly lower than that of *R. coptidis* extract (*P* < 0.01). Furthermore, the bacteriostatic activities of EPI^−^, PAL^−^, and (JAT + COL)^−^ were not significantly different to that of *R. coptidis* extract. Thus, BER and COP appear to be the main bacteriostatic constituents of *R. coptidis* extract with contributions to the bacteriostatic activity of 54.10% and 39.75%, respectively (Fig. 2d). 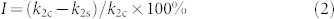

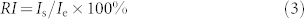
where *K*_2c_ is the growth rate constant of the second exponential growth phase of *S. dysenteriae* in the culture medium alone; *k*_2s_ is the growth rate constant of the second exponential growth phase of *S. dysenteriae* exposed to the test samples; *I*_s_ is the inhibition ratio of samples under evaluation; *I*_e_ is the inhibition ratio of *R. coptidis* extract (reference); and *RI* is the relative inhibition ratio.

### Constituent adding and chemical identification

**Figure 3:**
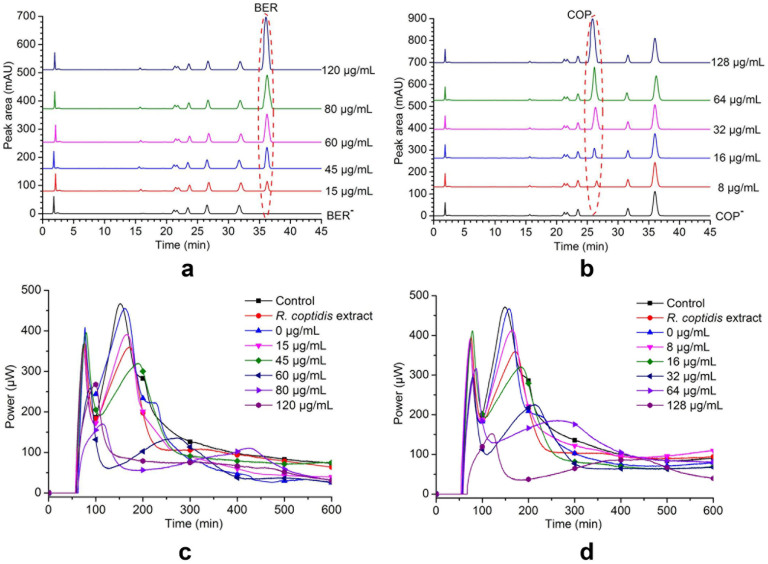
HPLC chromatograms following the adding of BER or COP and results of the bioassays. (a) HPLC profiles following BER added to the negative sample lacking constituent BER to final concentrations of 0, 15, 45, 60, 80, and 120 μg/mL. (b) HPLC profiles following COP added to the negative sample lacking constituent COP to final concentrations of 0, 8, 16, 32, 64, and 128 μg/mL. (c, d) heat flow power-time (HFP-*t*) curves of *S. dysenteriae* at 37°C exposed to *R. coptidis* extract or samples containing different concentrations of BER (c) or COP (d). *S. dysenteriae* in culture medium alone was used as the blank control. The concentration of *R. coptidis* extract was 200 μg/mL. The final concentrations of BER were 0, 15, 45, 60, 80, and 120 μg/mL. The final concentrations of COP were 0, 8, 16, 32, 64, and 128 μg/mL. The negative samples (BER^−^ and COP^−^) were prepared by removing BER or COP from the *R. coptidis* extract.

After confirming that BER and COP were the main bioactive constituents of *R. coptidis*, we added BER to its negative sample (BER^−^) to final concentrations of 0, 15, 45, 60, 80, and 120 μg/mL, which was equivalent to the relative content of BER in *R. coptidis* extract was (14.52%, based on the no-show detection process). HPLC was used to confirm the chemical compositions of the added samples. We also prepared added samples of COP at concentrations of 0, 8, 16, 32, 64, and 128 μg/mL, similar to the COP content in *R. coptidis* extract (5.3%, based on the no-show detection method). The HPLC profiles are shown in Fig. 3a, b and Supplementary Fig. S6.

### Levels of the active constituents

**Figure 4:**
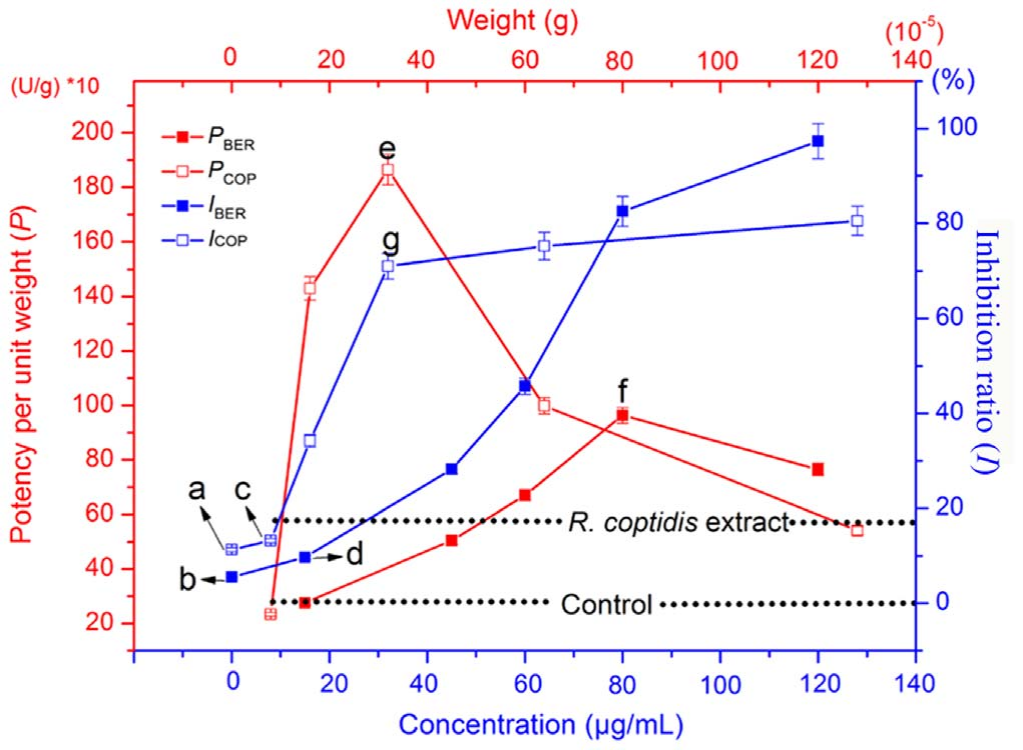
Relationship between the concentration of added BER/COP and *I*, and the potency of BER/COP on inhibiting the growth of *S. dysenteriae* at 37°C. The measurements of potency per unit weight and inhibition ratio were performed in triplicate and error bars represent standard error of the mean.

The bacteriostatic activity of samples adding BER or COP was assessed by microcalorimetry (Fig. 3c,d), and *I* and *RI* were calculated (Tables 1 and 2). The relationship between *RI* and BER or COP concentrations are shown in Fig. 4. To determine the correlation between the concentrations of active constituents in added samples and *I*, we calculated the change in *I* induced by the each concentration of active constituents (*P*) using equation (4): 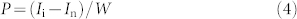
where *I*_n_ is the inhibition ratio of the negative sample lacking the targeted constituent; *I*_i_ is the inhibition ratio of the negative sample following the targeted constituent added; and *W* is the corresponding total concentration of the targeted constituent causing *I*_i_.

As illustrated in Fig. 4, the relative potencies of COP (i.e., *P*_COP_) and BER (i.e., *P*_BER_) were similar (red curves in Fig. 4). For both constituents, their relative potencies increased with increasing weight. However, the potency started to decrease when the weight passed a threshold level. Therefore, the peak values in the *P*_COP_ and *P*_BER_ curves (e and f in Fig. 4) are likely to show the greatest potencies. Therefore, the concentrations of COP and BER that showed the greatest efficiency were 32 μg/mL and 80 μg/mL, respectively. We calculated that the greatest efficiency of COP and BER is at the relative concentrations 14.45% and 31.92%, respectively.

The dose–response curves in Fig. 4 (blue curves) revealed some differences in *I*_COP_ and *I*_BER_. Points a and b in Fig. 4 correspond to the *RI*s of COP and BER, respectively. These values differed because the corresponding negative samples contained other constituents. Points c and d in Fig. 4 correspond to the *RI*s of 8 μg/mL COP and 15 μg/mL BER, respectively. At concentrations of COP and BER exceeding 8 μg/mL and 15 μg/mL, respectively, the *RI*s increased rapidly, and there was a significant difference between *I*_i_ and *I*_n_. We also found that the relative compositions of COP and BER which showed the best efficiency were 4.05% and 8.08%, respectively (Tables 1 and 2). The *RI* of COP did not increase markedly with further increases in concentrations after point g. Thus, the concentration of 32 μg/mL, corresponding to point g, might be the maximum dose of COP. By contrast, because there was no apparent plateau for *I*_BER_, we could not determine the maximum dose of BER. Although this finding might be due to synergistic effects of BER combined with other constituents of the negative samples, further studies are needed to confirm this possibility. By comparing the *I*_BER_ and *P*_BER_ curves, we found that *P*_BER_ decreased sharply after point f, but *I*_BER_ did not increase substantially. Accordingly, we speculate that the concentration of 80 μg/mL, which corresponds to point f, could be the maximum dose limit of BER. By comparing the relative compositions of the knocked in constituents (Table 1), we found that the relative composition of BER with the greatest efficiency was 31.92%.

## Discussion

Currently, the choice of QC indicators for herbal medicines is often subjective without forceful evidences^12^,^13^,^14^. Inspired by the discovery of functional genes, we put forward the strategy of constituent removing and adding according to the dual attributes of genetic information (genes) and chemical information (constituents) of organisms, hoping to promote the QC of herbal medicines. With this method, we found that the main bioactive constituents in *R. coptidis* that inhibited the growth of *S. dysenteriae* were BER and COP, with contributions to the bacteriostatic activity of 54.10% and 39.75%, respectively. By adding BER and COP into their negative samples, we found that the relative concentrations of BER and COP were 8.08%–31.92% and 4.05%–14.45%, respectively. We also found that BER alone did not fully account for the bacteriostatic activity of *R. coptidis*, although BER preparations are now widely used in clinical settings.

Although our strategy was inspired by gene knockout and knock-in, there are fundamental differences between these methods. In particular, gene knockout and knock-in involves gene recombination, and changes to the expression of genes through knockout, knock down, and silencing, which has dynamic features and can be expanded^19^,^20^,^21^,^22^,^23^. In terms of removing and adding of the constituents of an herbal medicine, there are five technical issues that need to be considered: (1) What constituent is being removed? (2) Was the removed constituent a single compound? (3) Does removal affect the structure of the targeted constituent? (4) How to add the constituent? (5) Are there any sensitive and high-throughput methods that can be used to assess the bioactivity? To deal with those issues in the study of constituent removing and adding of herbs, we used the convenient and reliable PTLCP to remove the bioactive constituents capable of inhibiting the growth of *S. dysenteriae*. Although JAT and COL were not separated completely, the result showed that the combination of them contributed little to the bacteriostatic activity of *R. coptidis* extract, which meant that the two constituents were not mainly active ones. Additionally, we performed HPLC and MS-MS to confirm that the removed constituents were not damaged, and there were almost no residual constituents in the negative samples. We also verified that the active constituents were added correctly. By using PTLCP, we were able to prepare sufficient quantities of samples. Although there are many methods available to assess bacteriostatic activity, we used a sensitive and high-throughput method called microcalorimetry to assess the bioactivity of the removed and added constituents.

A common characteristic of complex living organisms, such as herbal medicines, is that all of the constituents work together to produce an effect that is greater than the effects of the individual constituents. Unlike the traditional method of separating and then testing the activity of individual phytochemicals, we tried to avoid the limitations of reductionism in studies of complex systems without considering the interactions among constituents. Instead, we removed the targeted constituents from a complex system and then added them into the system to investigate the activity of samples. In this way, we ensure our conclusions can be used to establish a QS for herbs that is suitable for the clinical use and has guaranteed the clinical efficacy. Although there is a limitation of the strategy for its dependence on the separation of constituents, the combination of multiple techniques such as preparative high performance liquid chromatography and high speed counter current chromatography could be used to remove the targeted constituent^35^,^36^,^37^. Meanwhile, with the development of further researches on the constituent separation of herbal medicines, we believe that the strategy will also be improved better. Further studies that evaluate the synergistic and antagonistic properties of the bioactive constituents of herbs would aid the development of multicomponent drugs.

## Methods

### Materials

*R. coptidis* (*Coptis chinensis* Franch.) samples of about 5 years old were obtained using Good Agricultural Practice from Shizhu county, Chongqing province, China, in October 2010. All of the samples satisfied the standard for *R. coptidis* in the Chinese Pharmacopoeia and the Japanese Pharmacopoeia.

Reference compounds, including BER, PAL, COP, EPI, JAT, and COL were obtained from the National Institutes for Food and Drug Control (NIFDC) (Beijing, China); their purities were not less than 99.0% by high-performance liquid chromatography (HPLC).

*S. dysenteriae* (CMCC B 51252) was provided by the China Center for Type Culture Collection (Wuhan University, Wuhan, China). *S. dysenteriae* is the main pathogen causing dysentery. *R. coptidis* cures dysentery by inhibiting such pathogens. *S. dysenteriae* is frequently used to study the bioactivity of drugs used to treat diarrhea. Luria-Bertani (L.B.) culture medium was used in the bioassay and contained 5 g yeast extract, 5 g sodium chloride, and 10 g peptone in 1000 mL (pH = 7.2–7.4). The culture medium was sterilized at 121°C under 0.1 MPa for 30 min, and stored at 4°C until required.

Acetonitrile and methanol (chromatographic grade) were purchased from Fisher Chemicals (Pittsburgh, PA). Water was purified using a Milli-Q Water Purification System (Millipore, Bedford, MA). Silica Gel G preparative thin layer chromatography plates (PTLCP, 20 × 20 cm wide, 1 mm thick) were purchased from Yantai Chemical Industry Research Institute (Shandong, China). All other chemicals were purchased from Beijing Chemical Factory (Beijing, China) and were of analytical grade.

### Preparation of the Standard Solutions and the *R. coptidis* Extract

Standard solutions containing BER, PAL, COP, EPI, JAT, and COL were prepared by accurately weighing specific amounts of each substance and dissolving them in methanol in a volumetric flask. The concentrations of the standard solutions were as follows: BER 0.155 mg/mL, PAL 0.045 mg/mL, COP 0.032 mg/mL, EPI 0.035 mg/mL, JAT 0.016 mg/mL, and COL 0.018 mg/mL.

The *R. coptidis* extract was prepared as follows. The herb was first dried at room temperature and milled into a powder. Then, about 50 g of the powder was transferred to a flask containing 500 mL of purified water and mixed for 30 min. Reflux extraction was performed twice for 1.0 h each. The resulting solution was filtered while it was still hot and the filtrate was collected. Finally, the filtrate was evaporated to dryness and the solid residue was dried in a desiccator.

### Constituent removing

A silica Gel G PTLCP was used to separate and obtain the constituents of *R. coptidis*. The water extract of *R. coptidis*, and standard solutions of BER, PAL, COP, EPI, JAT, and COL were spotted onto the same plate, and developed in a mixture of toluene, ethylacetate, methanol, isopropanol and ammonia water (6:3:1.5:1.5:0.5, *v*/*v*). After observing the plate under daylight, the bands corresponding to the relative retention values of each reference compound were retrieved. These procedures were repeated. Then, the merged silica chips were extracted with methanol and the suspensions were filtered and centrifuged. The supernatants were collected and evaporated to dryness for further chemical analysis and for use in bioassays to identify the active constituents in *R. coptidis*.

### Constituent adding

After identifying the bioactive constituents of *R. coptidis*, we applied the added procedure. In this phase, the active constituents were dissolved in L.B. culture medium to the required concentrations. Then, the dissolved constituent was added into the negative sample (prepared in L.B. culture medium) without the corresponding constituent. Then, we performed chemical analyses to characterize the constituents. Finally, microcalorimetry was performed to determine the bioactivity of different amounts of each active constituent to determine their levels in the herbal extract.

### Chemical analysis

HPLC was performed to determine the levels of the active constituents in *R. coptidis* extract, evaluate the changes in the chemical constituents following removing and adding, as follows. HPLC was performed using an Agilent 1200 HPLC system (Agilent Technologies, Santa Clara, CA). Chromatographic separation and detection of samples were performed on a Kromasil™ C_18_ column (250 mm × 4.6 mm, 5 μm) at a column temperature of 30°C with a flow rate of 0.6 mL/min using a solution of acetonitrile/water (0.05 mol/L potassium dihydrogen phosphate) (50:50, *v*/*v*). Then, sodium dodecyl sulfate was added to a concentration of 0.4 g/100 mL of solution (pH = 4.0). The standard solutions, *R. coptidis* extract, active constituents obtained by PTLCP separation, and the added samples were filtered through a 0.22 μM Millipore membrane (Carrigtwohill, Co. Cork, Ireland) and injected into the HPLC system for chemical analysis.

### Bioactivity assay

The bioactivity experiments were performed using the ampoule method at 37°C with a 3114/3236 TAM air isothermal microcalorimeter (Thermometric AB, Jarfalla, Sweden). *S. dysenteriae* were inoculated into 100 mL of L.B. culture medium at an initial density of 1 × 10^6^ colony forming units (CFU) per mL. Then, 10 mL of the bacterial suspension was added to sterilized 20-mL glass ampoules. The *R. coptidis* extract, removed samples, and added samples were then added to the bacterial suspension. Each ampoule was sealed and placed in an eight-channel calorimeter. When the temperature of the ampoules reached 37°C, heat flow power-time (HFP-*t*) curves were recorded for each sample until the values returned to baseline. Each experiment was repeated three times. All data were continuously recorded using PicoLog TC-80 software (TA Corporation, New Castle, USA).

Microcalorimetry data are repeatable and the method provides real-time, online, dynamic information to characterize the amount of heat produced by organisms. We measured the following thermokinetic parameters: the growth rate constants of the first and second exponential phases (*k*_1_ and *k*_2_), the heat flow power (HFP) of the first and the second highest peaks (*p*_1_ and *p*_2_), and the appearance time of the first and second highest peaks (*t*_1_ and *t*_2_) from the HFP-*t* curves.

### Data analysis

Quantitative thermokinetic parameters were analyzed using Origin 8.5 software (OriginLab Company, Northampton, USA) to plot the HFP-*t* curves, and calculate the thermokinetic parameters.

Principal component analysis (PCA) is a data reduction technique that can be used to extract data, remove redundant information, highlight hidden features, and visualize the relationships among numerous variables with a small number of underlying factors (principal components or PCs) without losing crucial information^38^. Therefore, we performed PCA to identify the main parameters derived from the HFP-*t* curves.

We also applied analysis of variance (ANOVA) to test for differences in the bioactivity of different samples, and identify the active components. This procedure calculates whether there is a significant difference between the observed value (i.e., *F* value) and the corresponding probability (*P* value).

## Author Contributions

D.Y., J.L. and C.Z. conducted the experiments. D.Y., J.L. and Y.X. wrote the manuscript and prepared figures. J.L., Y.H., H.Q. and L.Q. conducted sample collection and data analysis. C.P., X.S., J.L., R.W., C.J. and Y.L. provide materials and data. D.Y. and X.X. conceived the study. All authors reviewed the manuscript.

## Supplementary Material

Supplementary Information Supporting Information

## Figures and Tables

**Figure 1:**
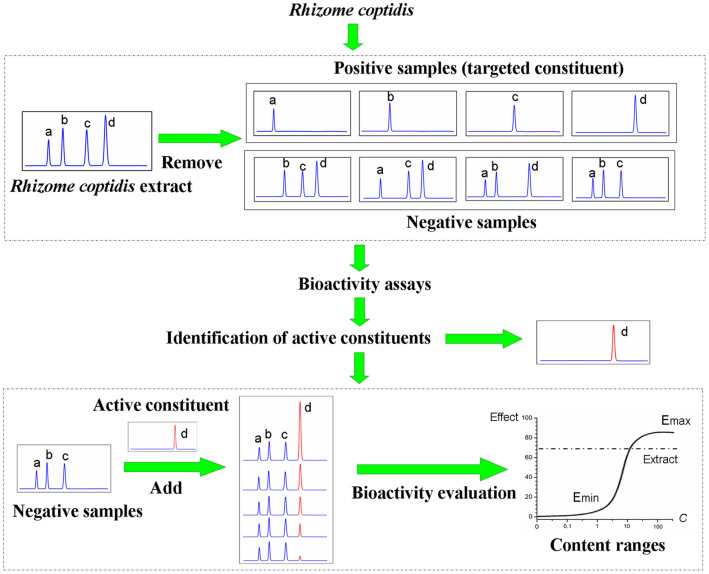
The strategy used to discover the active constituents and their levels in *Rhizome coptidis*.

**Table 1:** Thermokinetic characteristics of *R. coptidis* (200 μg/mL) and samples containing different concentrations of BER on *S. dysenteriae* growth at 37°C (*n* = 3, Mean ± *SD*)

*C* (μg/mL)	*k*_1_ (min^−1^)	*k*_2_ (min^−1^)	*t*_1_ (min)	*p*_1_ (μW)	*t*_2_ (min)	*p*_2_ (μW)	*I* (%)	*P* (U/g)	*W* (%)
Control	0.107 ± 0.011	0.021 ± 0.007	74.0 ± 1.1	369.2 ± 2.1	152.0 ± 1.6	467.0 ± 3.0	0.00	–	–
*R. coptidis*	0.090 ± 0.008	0.017 ± 0.003	76.0 ± 1.2	371.4 ± 2.3	170.3 ± 1.9	359.4 ± 3.7	16.38	–	–
0§	0.109 ± 0.010	0.020 ± 0.002	77.3 ± 0.6	408.4 ± 1.5	161.0 ± 1.4	455.2 ± 3.5	5.51	–	0.00
15§	0.097 ± 0.005	0.018 ± 0.003	74.7 ± 0.5	358.0 ± 2.6	166.3 ± 1.3	391.1 ± 3.2	9.65	27.6	8.08
45§	0.082 ± 0.009	0.015 ± 0.004	80.0 ± 0.9	395.5 ± 2.8	189.0 ± 1.9	320.2 ± 2.9	28.23	50.5	20.87
60§	0.079 ± 0.007	0.011 ± 0.003	88.0 ± 1.3	258.1 ± 2.0	271.3 ± 2.3	136.1 ± 1.3	45.73	67.0	26.02
80§	0.032 ± 0.004	0.005 ± 0.002	114.0 ± 1.4	170.8 ± 1.6	424.3 ± 3.2	111.1 ± 2.2	82.55	96.3	31.92
120§	0.023 ± 0.005	0.003 ± 0.001	424.3 ± 2.1	268.5 ± 2.4	185.0 ± 2.9	81.7 ± 2.1	97.32	76.5	41.29

*C*: concentration (μg/mL), *k*_1_ and *k*_2_: the growth rate constants in the first and second exponential phases, *t*_1_ and *t*_2_: the appearance time of the first and second highest peaks, *p*_1_ and *p*_2_: the heat flow power (HFP) of the first and the second highest peaks, *I*: inhibition ratio in stage *k*_2_, *P*: relative potency of BER, *W*: content of BER in the added sample, *W* = *W*_BER_/(*W*_BER_ + *W*_BER_^−^) × 100%, where *W*_BER_ is the added weight of BER, *W*_BER_^−^ is the weight of *R*. *coptidis* extract (200 μg/mL) with BER removed [*W*_BER_^−^ = (1–the content of BER in the *R*. *coptidis* extract) × 200 μg/mL]. ^§^Concentration of BER in the negative sample. The above measurements were performed in triplicate and error bars represent standard error of the mean.

**Table 2:** Thermokinetic characteristics of *R*. *coptidis* (200 μg/mL) and samples containing different concentrations of COP on *S*. *dysenteriae* growth at 37°C (*n* = 3, Mean ± *SD*)

*C* (μg/mL)	*k*_1_ (min^−1^)	*k*_2_ (min^−1^)	*t*_1_ (min)	*p*_1_ (μW)	*t*_2_ (min)	*p*_2_ (μW)	*I* (%)	*P* (U/g)	*W* (%)
Control	0.107 ± 0.015	0.020 ± 0.002	73.7 ± 1.3	371.4 ± 3.6	150.7 ± 1.1	470.0 ± 3.3	0.00	–	–
*R. coptidis*	0.090 ± 0.009	0.017 ± 0.001	75.7 ± 1.0	376.5 ± 2.2	170.0 ± 2.3	359.5 ± 2.2	17.96	–	–
0§	0.101 ± 0.010	0.018 ± 0.003	73.7 ± 0.5	387.2 ± 1.1	158.3 ± 0.5	467.6 ± 3.0	11.33	–	0.00
8§	0.096 ± 0.008	0.016 ± 0.004	73.7 ± 0.9	391.2 ± 2.4	165.7 ± 0.9	413.9 ± 4.1	13.20	23.4	4.05
16§	0.079 ± 0.005	0.013 ± 0.002	78.7 ± 0.7	411.8 ± 1.5	182.3 ± 1.4	321.0 ± 3.4	34.20	142.9	7.79
32§	0.065 ± 0.002	0.006 ± 0.001	79.3 ± 1.5	296.7 ± 3.2	214.3 ± 1.9	225.9 ± 2.6	71.00	186.5	14.45
64§	0.054 ± 0.007	0.005 ± 0.002	87.0 ± 1.9	317.5 ± 3.5	262.0 ± 1.7	186.2 ± 1.7	75.22	99.8	25.26
128§	0.046 ± 0.003	0.004 ± 0.001	120.0 ± 2.4	152.4 ± 2.8	442.7 ± 3.1	88.6 ± 1.8	80.52	54.1	40.33

*C*: concentration (μg/mL), *k*_1_ and *k*_2_: the growth rate constants in the first and second exponential phases, *t*_1_ and *t*_2_: the appearance time of the first and second highest peaks, *p*_1_ and *p*_2_: the heat flow power (HFP) of the first and the second highest peaks, *I*: inhibition ratio in stage *k*_2_, *P*: relative potency of COP, *W*: content of COP in the added sample, *W* = *W*_COP_/(*W*_COP_ + *W*_COP_^−^) × 100%, where *W*_COP_ is the added weight of COP, *W*_COP_^−^ is the weight of *R*. *coptidis* extract (200 μg/mL) with COP removed [*W*_COP_^−^ = (1–the content of COP in the *R*. *coptidis* extract) × 200 μg/mL]. ^§^Concentration of COP in the negative sample. The above measurements were performed in triplicate and error bars represent standard error of the mean.
