# Supplementary material for: Promotion of quality standard of herbal medicine by constituent removing and adding
Source: Sci Rep. 2014 Jan 13;4:3668. doi: 10.1038/srep03668 (PMC3888971; doi:10.1038/srep03668)
Supplement: Supplementary Information — Supporting Information [file srep03668-s1.pdf]

Supporting Information

Promotion of quality standard of herbal medicine by constituent removing and adding

Dan Yan[1,2,7], Junxian Li[1,7], Yin Xiong[1,3,7], Congen Zhang[1], Jiaoyang Luo[4], Yumei Han[5], Ruiling Wang[1], Hong Qian[5], Jiangyu Li[1], Lingling Qiu[1], Cheng Peng[6], Yuling Lin[4], Xueai Song[1] & Xiaohe Xiao[1*]

[1] China Military Institute of Chinese Materia Medica, Military 302 Hospital, Beijing 100039, China, [2] National Center of Biomedical Analysis, Beijing 100850, China, [3] College of Traditional Chinese Pharmacy, Beijing University of Chinese Medicine, Beijing 100102, China, [4] Institute of Medicinal Plant Development, Chinese Academy of Medical Sciences, Beijing 100094, China, [5] Beijing Physical Examination Center, Beijing 100077, China, [6] Chengdu University of Traditional Chinese Medicine, Chengdu 610075, China

[*]Correspondence and requests for materials should be addressed to X.X.

(E-mail: pharmsci@126.com, Tel.: 0086-10-66933325, Fax: 0086-10-66933325)

[7]These authors contributed equally to this work.

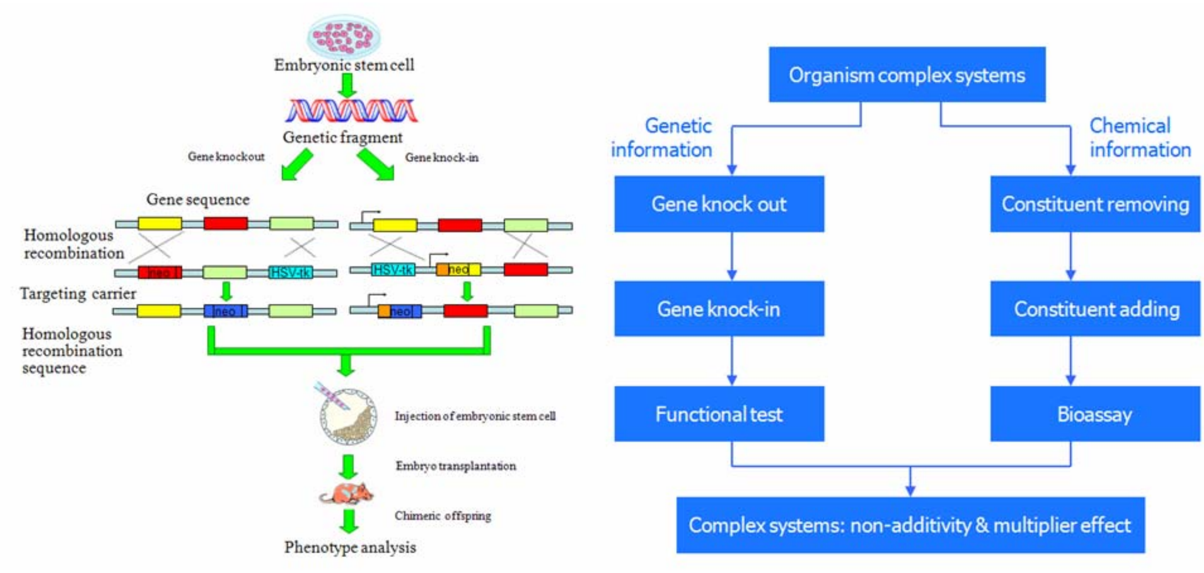

Supplementary Fig. S1 (a) Illustration of the removing and adding strategy used to examine the roles of functional genes[1-5]. (b) Comparison of the strategies used to evaluate functional genes and active constituents in the complex organisms. Images by Lingling Qiu and Dan Yan.

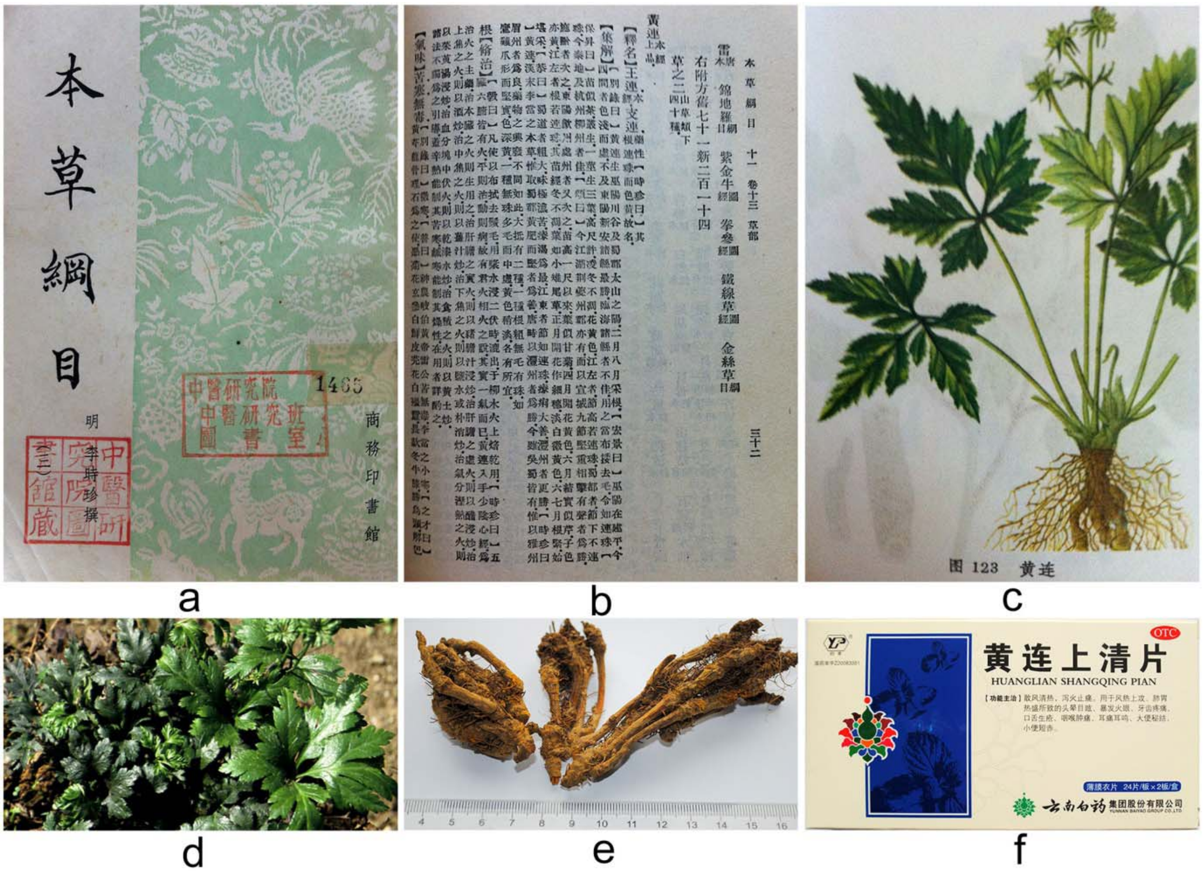

Supplementary Fig. S2 Stages in the medicalization of *Rhizome coptidis* from a plant resource to a herbal medicine. *Rhizome coptidis* (*R. coptidis*) is an important herbal medicine that has been used in Asia, Africa, and Latin America for thousands of years. It was recorded that this herb was cultivated in the Tang Dynasty (742 AD) in China and in the Nara Age (710 AD) in Japan. *R. coptidis* has been used as a herbal medicine to treat bacterial diarrhea, constipation, diabetes, and other major diseases[6-10]. Photographs by Yin Xiong and Yuling Lin.

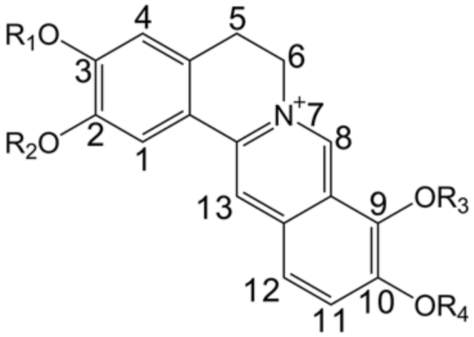

	R_1	R_2	R_3	R_4
berberine (BER)	—CH_2—		CH_3	CH_3
palmatine (PAL)	CH_3	CH_3	CH_3	CH_3
coptisine (COP)	—CH_2—		—CH_2—	
epiberberine (EPI)	CH_3	CH_3	—CH_2—	
jateorrhizine (JAT)	H	CH_3	CH_3	CH_3
columbamine (COL)	CH_3	CH_3	CH_3	H

Supplementary Fig. S3 The structures of constituents removed from *R. coptidis* extract. Constituents removed from *R. coptidis* extract include berberine (BER), palmatine (PAL), coptisine (COP), epiberberine (EPI), jateorrhizine (JAT), and columbamine (COL). All of them are protoberberine alkaloids, belong to isoquinoline type alkaloids, of which the difference results from groups bound to C2, C3, C9 and C10.

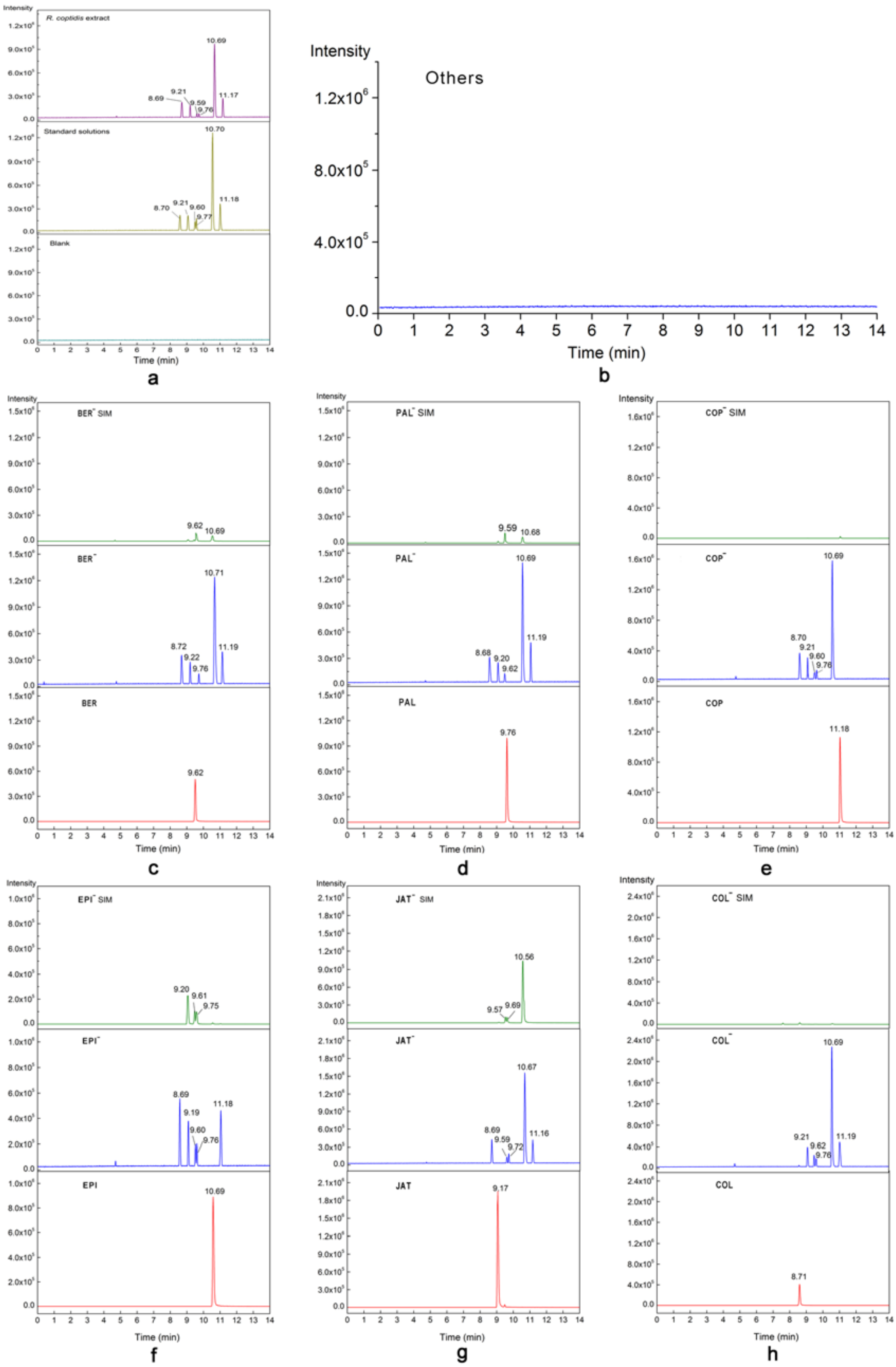

Supplementary Fig. S4 UPLC/Q-TOF-MS chromatograms obtained in the positive ion mode for the samples removed. SIM: single ion monitoring (SIM) analysis based on the molecular weight of each constituent. Peaks corresponding to the removed constituents were not detected in the negative samples or in single ion monitoring analysis.

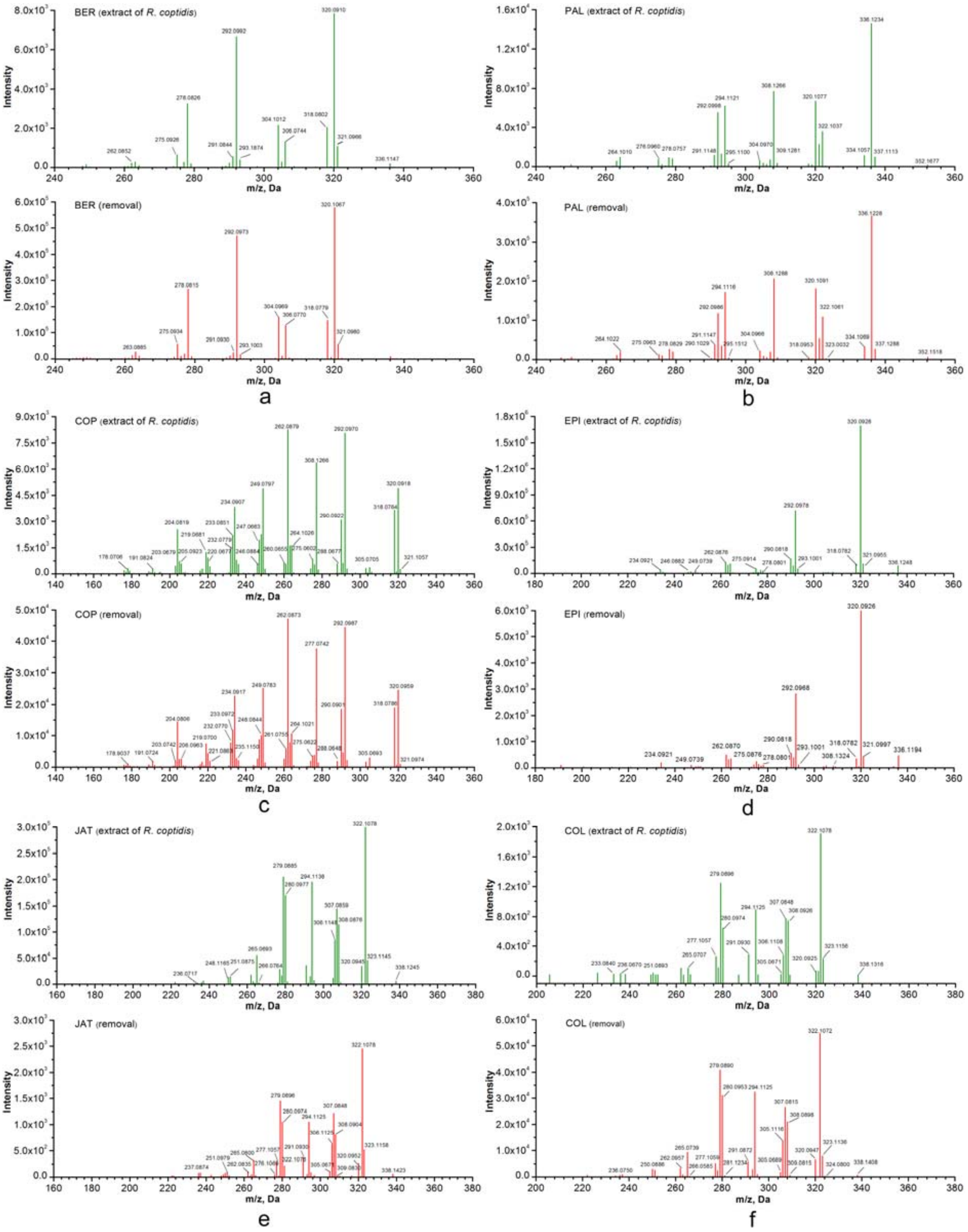

Supplementary Fig. S5 MS-MS spectra. Panels a–f represent the product ion spectra for the molecular peaks at *m/z* 338.1392, 338.1392, 336.1236, 336.1236, 352.1549, and 320.0923, respectively. Spectras are shown for the positive samples in the *R. coptidis* extract, and after removing of each constituent. This figure shows that the same fragment ions were detected in the removed samples and in the *R. coptidis* extract.

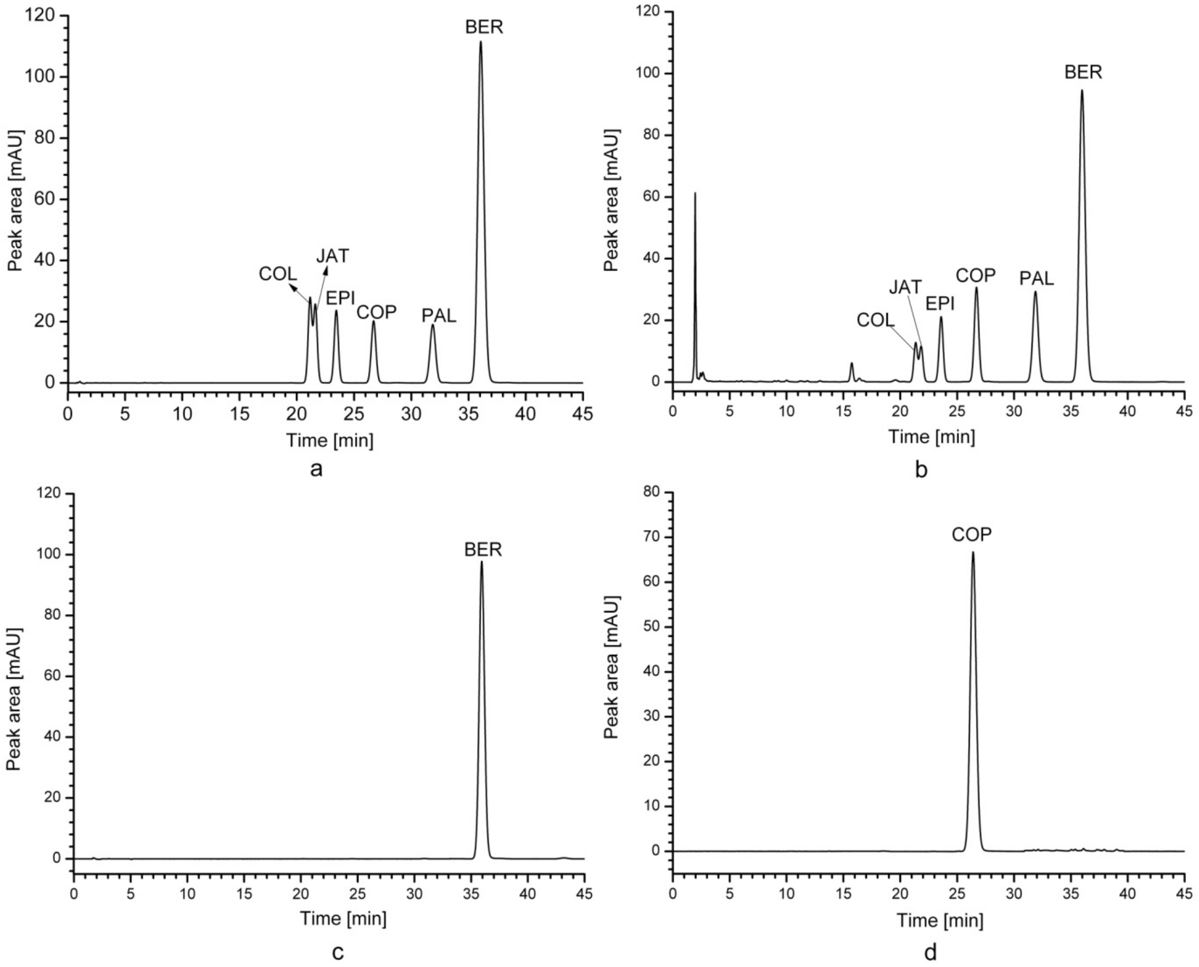

Supplementary Fig. S6 HPLC chromatograms of samples following BER or COP added. (a) Mixed standard solutions, (b) *R. coptidis* extract, (c) BER removed, (d) COP removed.

Supplementary Table S1 Thermokinetic characteristics of *R. coptidis* extract, the removed constituents and the corresponding negative samples on *S. dysenteriae* growth at 37°C (n=3, Mean±SD)

Sample	k_1 (min^{-1})	k_2 (min^{-1})	t_1 (min)	p_1 (μW)	t_2 (min)	p_2 (μW)	I (%)	RI (%)
Control	0.103±0.012	0.020±0.002	76.3±2.3	265.9±3.6	158.7±2.2	470.4±3.5	–	–
R. coptidis	0.014±0.012	0.003±0.001	95.3±3.6	287.9±2.2	271.3±0.7	170.1±2.7	82.21	100.00
BER^{+}	0.032±0.002	0.011±0.001	96.3±2.1	358.1±0.8	285.3±1.3	257.6±1.9	43.92	53.42
BER^{-}	0.047±0.005	0.014±0.002	77.3±1.2	264.5±3.4	183.7±2.4	221.6±2.3	30.27	36.82
COP^{+}	0.045±0.004	0.014±0.003	87.3±1.5	391.1±2.1	190.7±2.2	349.5±2.4	30.52	37.13
COP^{-}	0.031±0.003	0.011±0.002	88.7±1.7	266.0±1.3	223.0±0.4	268.5±2.7	43.56	52.99
$(COL + JAT)^{+}$	0.068±0.001	0.019±0.001	75.3±1.1	328.8±1.4	161.3±1.8	451.3±1.0	2.71	3.30
$(COL + JAT)^{-}$	0.018±0.002	0.004±0.001	96.3±0.9	308.1±2.4	285.3±2.7	207.6±0.6	79.60	96.83
PAL^{+}	0.061±0.004	0.019±0.004	63.3±0.6	244.3±3.8	147.3±3.2	444.8±1.4	3.58	4.35
PAL^{-}	0.018±0.002	0.004±0.002	68.3±1.8	229.7±1.1	229.0±4.1	182.2±3.3	78.48	95.46
EPI^{+}	0.064±0.002	0.019±0.001	66.0±1.9	296.9±0.7	146.3±1.2	454.8±3.0	2.10	2.55
EPI^{-}	0.017±0.003	0.004±0.001	73.3±2.5	224.7±1.3	234.0±2.4	177.2±3.2	80.21	97.57
Alkaloids[‡]	0.012±0.002	0.002±0.000	83.3±1.8	259.0±1.7	252.3±2.0	189.5±3.4	91.51	111.32
Others	0.071±0.005	0.020±0.003	56.3±1.4	272.1±2.9	138.3±1.9	473.1±1.7	-4.45	-5.41

k_1 and k_2: the growth rate constants in the first and second exponential phases, t_1 and t_2: the appearance time of the first and second highest peaks, p_1 and p_2: the heat flow power (HFP) of the first and the second highest peaks, I: inhibition ratio in stage k_2, RI: relative inhibition ratio with *R. coptidis* extract as the reference. [‡]The alkaloids are BER, COP, COL, JAT, PAL, and EPI. The above measurements were performed in triplicate and error bars represent standard error of the mean.

Supplementary Methods

Q-TOF analysis conditions[11]

Samples were analyzed using ACQUITY UPLC and Xevo G2 Q-TOF MS systems (Waters Micromass, Manchester, UK) equipped with an electrospray ion source and a hybrid quadrupole time-of-flight (Q-TOF) mass spectrometer. UPLC analysis was conducted on a Waters Acquity UPLC BEH C_{18} column (2.1 mm × 50 mm, 1.7 μm). The mobile phases were solvent A (10 mmol/L ammonium acetate/0.05% formic acid in water), solvent B (80:20 methanol:acetonitrile), with the following elution gradient: 95%–90% A at 0–2 min, 90%–80% A at 2–5 min, 80%–70% A at 5–10 min, 70%–60% A at 10–14 min, and 60%–0% A at 14–15.8 min, The flow rate was held at 0.30 mL/min and the column temperature was 45°C.

ESI-MS spectra were acquired in the positive ion mode. For full-scan MS, the spectra were recorded in the range of m/z 50–800. The ESI conditions were as follows: capillary voltage, 3.0 kV; source block temperature, 100°C; desolvation temperature, 350°C; sample cone voltage, 40 V; and desolvation gas flow, 700.0 L/H. The system was automatically calibrations from m/z 50 to 1200 with sodium formate solution. For automated mass measurement, a lock-spray probe was used, and the external reference (0.2 ng/mL leucine enkephalin) was infused at a flow rate of 5 μL/min via the lock-spray interface, generating reference ions at 556.2771 Da and 278.1141 Da. The lock-spray scan time was 0.5 s with an interval of 10 s; data were averaged over three scans. A collision energy ramp ranging from 35 to 55 eV was optimized for MS/MS analysis, using a scan time of 0.3 s and an interval of 0.014 s.

1. Lewandoski, M. Conditional control of gene expression in the mouse. *Nat. Rev. Genet.* **2,** 743-755 (2001).
2. Rudolph, U., Crestani, F. & Möhler, H. $GABA_A$ receptor subtypes: dissecting their pharmacological functions. *Trends Pharmacol. Sci.* **22,** 188-194 (2001).
3. Zwaka, T. P. & Thomson, J. A. Homologous recombination in human embryonic stem cells. *Nat. Biotechnol.* **21,** 319-321 (2003).
4. Manis, J. P. Knock out, knock in, knock down—genetically manipulated mice and the Nobel Prize. *N. Engl. J. Med.* **357,** 2426-2429 (2007).
5. Tong, C., Li, P., Wu, N. L., Yan, Y. & Ying, Q.L. Production of p53 gene knockout rats by homologous recombination in embryonic stem cells. *Nature* **467,** 211-213 (2010).
6. Cheung, F. TCM: made in China. *Nature* **480,** S82-S83 (2011).
7. Bova, S., Padrini, R., Goldman, W., Berman, D. & Cargnelli, G. On the mechanism of vasodilating action of berberine: possible role of inositol lipid signaling system. *J. Pharmacol. Exp. Ther.* **261,** 318-323 (1992).
8. Li, C.Y., Tsai, S.I., Damu, A.G. & Wu, T.S. A rapid and simple determination of protoberberine alkaloids in Rhizoma Coptidis by ^{1}H NMR and its application for quality control of commercial prescriptions. *J. Pharmaceut. Biomed.* **49,** 1272-1276 (2009).
9. Sun, C. & Liu, H. Application of non-ionic surfactant in the microwave-assisted extraction of alkaloids from *Rhizoma Coptidis*. *Anal. Chim. Acta* **612,** 160-164 (2008).
10. Tan, B., Ma, Y., Shi, R. & Wang, T. Simultaneous quantification of three alkaloids of *Coptidis Rhizoma* in rat urine by high-performance liquid chromatography: application to pharmacokinetic study. *Biopharm. Drug Dispos.* **28,** 511-516 (2007).
11. Chen J. H. *et al*. Analysis of alkaloids in *Coptis chinensis* Franch by accelerated solvent extraction combined with ultra performance liquid chromatographic analysis with photodiode array and tandem mass spectrometry detections. *Anal. Chim. Acta* **613,** 184-195 (2008).
